# Advances in telemedicine implementation for preoperative assessment: a call to action

**DOI:** 10.1186/s44158-024-00172-4

**Published:** 2024-06-05

**Authors:** Elena Giovanna Bignami, Michele Berdini, Matteo Panizzi, Valentina Bellini

**Affiliations:** Anesthesiology, Critical Care and Pain Medicine Division, Department of Medicine and Surgery, University of Parma, Viale Gramsci 14, Parma, 43126 Italy

**Keywords:** Telemedicine, Artificial Intelligence, Preoperative Assessment, Consensus, Perioperative Medicine

Telemedicine uses communication technologies to provide remote health care services, allowing physicians to provide remote consultations, treatments and monitoring. It is an emerging and crucial element in today’s healthcare landscape, with progressive diffusion in many areas of the world [1] (Table 1). Preoperative teleconsultation should represent a complementary option to the traditional in-person medical examination as outlined by the Italian Code of Medical Ethics and National and European guidelines.

**Table 1:** Overview of key studies

Kamdar NV et.al	Illustrates the execution of a preoperative anesthesia evaluation via telemedicine at an academic medical center, resulting in high patient satisfaction, cost savings, and no rise in day-of-procedure case cancellations
Aldawoodi NN et.al	Shows that conducting preanesthesia evaluations through telemedicine leads to savings in time, distance, and finances, all while maintaining consistent day-of-surgery cancellation rates
Hayasaka T et.al	In this study deep learning has been applied to classify intubation challenges, using an AI model with high accuracy and sensitivity
Wong DT et.al	In this study telemedicine technology has been employed to predict mouth opening, Mallampati score, neck movement, thyromental distance, neck movement and to perform a heart and lungs assessment. Devices such as airway camera and digital stethoscope were utilized
Bhanvadia RR et.al	Examines the safety of exclusively utilizing telehealth for preoperative consultations preceding minimally invasive urologic procedures
Morau E et.al	Illustrates how Pre-anesthesia teleconsultation (PATC) is not inferior to pre-anesthesia consultation (PAC) for preoperative patient evaluation and how it may be a valid alternative to it
Applegate RL et.al	This article explores the impact of telemedicine pre-anesthesia evaluation on perioperative processes, highlighting patient satisfaction and time and cost saving benefits
Khera KD et.al	The aim of this study was to identify patient-related factors where a face to face (FTF) evaluation is indicated, over telemedicine evaluation

## Advantages

**Fig. 1:**
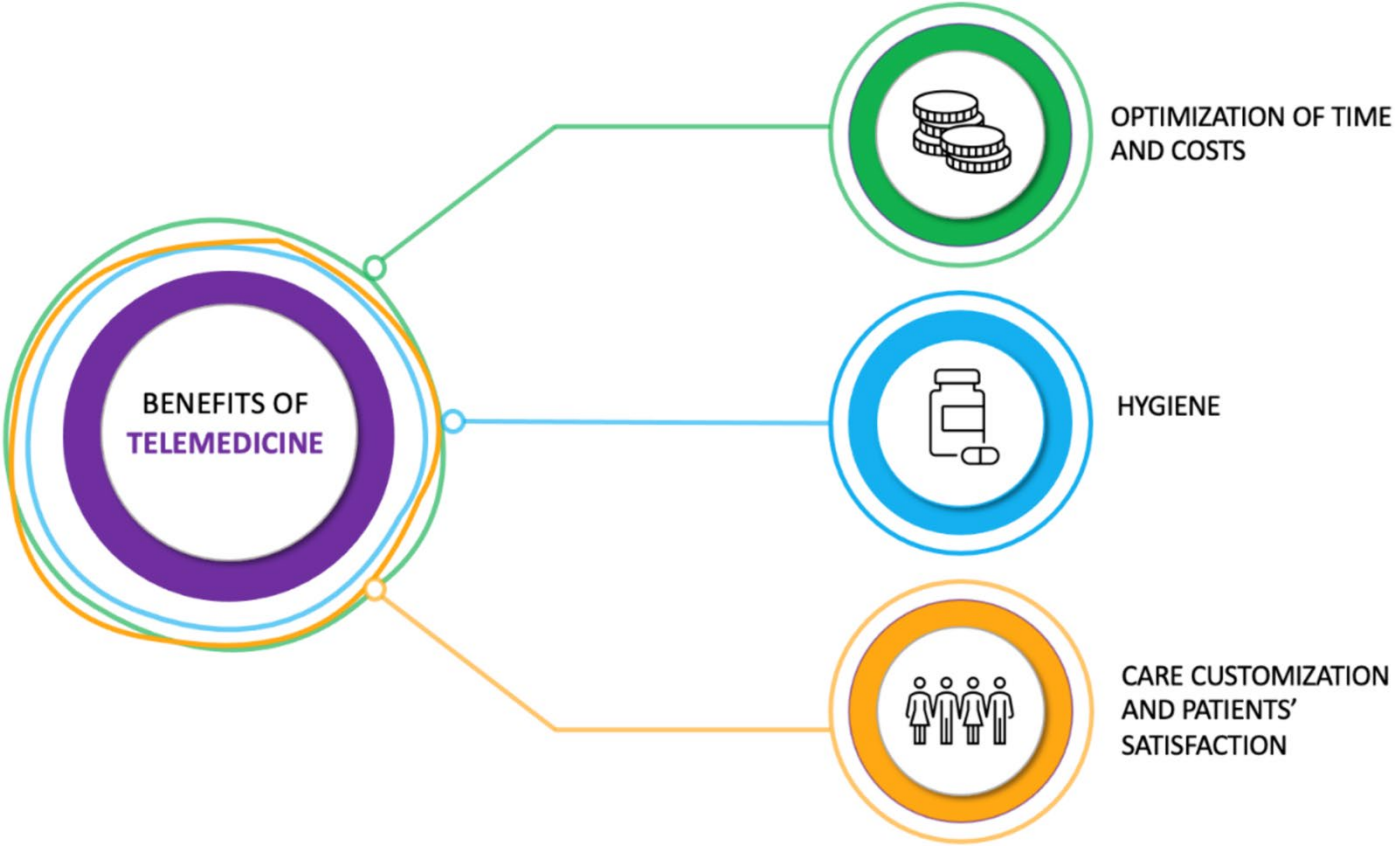
Benefits of preoperative medical examination conducted by telemedicine

Preoperative televisit offers significant advantages in terms of hygiene, care customization, time and costs optimization (Fig. 1). Moreover, it reduces the costs associated with transportation and lodging and the time spent travelling, allowing patients to participate from any comfortable location. It is also known that avoiding contact between patients and healthcare staff reduces the risk of transmission of respiratory viruses and their healthcare burden.

## Challenges and outstanding problems

### Universal accessibility

Current evidence suggests that the implementation of telemedicine in the preoperative visit has a rate of reduction in cancellations and delays of surgeries overlapping with the one of the live visit [2–4]. For example, the likelihood of cancellations due to mobility problems or logistical difficulties it is decreased because patients do not have to travel to healthcare facilities. To this end, telemedicine needs as its basis a reliable information system accessible to the entire population, regardless of the socioeconomic status, level of educational or disabilities of users.

In this perspective, challenges would arise from the variety of devices used by patients to participate in visits, with different operating systems and hardware specifications, as well as the heterogeneity of the Internet connection at their disposal. In addition, obstacles would arise from the usability of the software platforms: patients with limited digital skills or disabilities might find it difficult to use them without an intuitive and user-friendly interface or the support of a caregiver.

### Informed consent and cyber security

The transmission and understanding of the information required for informed consent depends on the efficiency of the system. Telemedicine platforms must have high-quality audio/video features with informative digital material easily accessible, but the most important aspects are privacy and cyber security. Some of the most relevant risk in this field are Remote work security, Endpoint device management, Human factors, Lack of security awareness, Limited budget and health care services delivery without disruption [5]. Blockchain technology thanks to its immutability, cryptographic security, and transparency, can address the issue of storing, transmit and process patient’s health data [6]. Thanks to decentralization, it can limit the security issues related to the management of a Local Area Network. The introduction of smart contracts allows a deeper control over data access depending on clinician privilege level and patient-controlled authorization.

For example, Informed Consent may be obtained via on-chain forms and signed both by clinicians and patient through they own private keys and stored securely on-chain and the implementation of Zero Knowledge (zk) blockchain rollups would allow an off-chain personal data storage leaving on-chain just the validation of data/signature [7].

This off-chain storage would allow the deletion of personal data exposing on-chain only the signature and thus making blockchain technology compliant with GDPR’s ‘right to be forgotten’ principle, which not possible if data are stored on-chain because of its immutability.

### Identity verification, digital signature and outcome tracking

Another major challenge is verification of patient identity: a possible solution may be the use of platforms with electronic signature features that comply with local regulations. This would ensure that informed consent is valid and legally binding. In Italy, for example, one of the alternatives that could be explored is SPID (Sistema Pubblico di Identità Digitale).

A step further: this objective can be achieved again through blockchain by creating a non-fungible token (NFT), a digital certificate that attests to the uniqueness, authenticity, and univocal ownership of a physical or digital object and all the information contained in it. A tokenized digital identity stored on blockchain would certify patient’s and clinician’s identity and signature verification. This NFT may also contain all patient’s past information such as habits, risk factors, current functional status, and may generate outcome trajectories based on them [8].

### Virtual objective examination

The limited ability to perform thorough physical examinations is an element that may raise uncertainties in preoperative assessment. However, the use of personal video-communication platforms is now a widely adopted solution, especially in the post-pandemic setting. In addition, with regard to anesthesiologic telemedicine, it should be noted that technologies for cardiopulmonary and airway assessment, crucial aspects of the assessment itself, are already in use and constantly improving [9, 10].

Despite the obvious limitations, the literature shows that the sensitivity between virtual and traditional live preoperative examination does not differ significantly, highlighting the potential of telemedicine as an effective tool for anesthesiologic evaluation [11–14].

### Eligibility of patients

Literature suggests face-to-face preoperative visits for patients over 65, with significant comorbidities (e.g., diabetes), or on 7 + medications, potentially needing additional preoperative examinations after anesthesiologic evaluation [15]. Although this might suggest that patients with higher ASA score need an in-person visit, some authors found no significant differences in the rate of procedure cancellation among patients with ASA score between 1 and 4, regardless of whether the meeting was scheduled in virtual or in-person mode. Even in high risk surgery, such as cardiac one, it has been effectively utilized achieving a safety profile comparable to the conventional physical consultations without recording any increase in surgical cancellations or morbidity rates. Even if this may suggest the feasibility of using televisit for selected ASA III or IV patients, most studies focused on patients with ASA score 1 or 2. For this reason further research are needed on patients with higher scores to clarify its safety. Certainly, the diversity and complexity of healthcare systems around the world imply that each reality has its own specificities and distinctive characteristics. Therefore, identifying criteria for inclusion and exclusion of suitable patients requires in-depth analysis contextualized to local peculiarities.

## Conclusions

The literature on this topic is still limited but telemedicine in anesthesiology continues to develop and innovative approaches are adopted. These challenges should be effectively addressed, ensuring an increasing level of integration between traditional consultations and telemedicine visits.

To achieve the result, the collaboration of a multidisciplinary team is essential to ensure an efficient and coordinated telematic process (Table 2) and the problem of standardization remains open because of the geographically different context an resources availability. For this reason, the creation of a consensus task force emerges as an essential step, with the aim of minimizing the risk of significant errors and promoting evidence-based clinical practice.

**Table 2:** Stakeholders and responsibilities of the multidisciplinary team

STAKEHOLDERS	RESPONSIBILITIES
Physician	Conducting television and clinical counseling Obtaining informed consent Prescribing clinical examinations
Legal staff	Ensuring regulatory compliance Management of legal issues Dispute management
Computer engineer	Platform development and implementation Cybersecurity and privacy protection Cyber data management
Computer technician	Technology support during sessions Software configuration and installation
Administrative staff	Documentation management Planning and coordination of visits
Data scientist	Data analysis, processing and interpretation Protocol optimization Cybersecurity and privacy protection

## Data Availability

No datasets were generated or analysed during the current study.

## References

[1] Bignami E, Lanza R, Cussigh G, Bellini V (2023). New technologies in anesthesia and intensive care: take your ticket for the future. J Anesth Analg Crit Care.

[2] Kamdar NV, Huverserian A, Jalilian L, Thi W, Duval V, Beck L, Brooker L, Grogan T, Lin A, Cannesson M (2020). Development, Implementation, and Evaluation of a Telemedicine Preoperative Evaluation Initiative at a Major Academic Medical Center. Anesth Analg.

[3] Zhang K, Rashid-Kolvear M, Waseem R, Englesakis M, Chung F (2021). Virtual preoperative assessment in surgical patients: A systematic review and meta-analysis. J Clin Anesth.

[4] Aldawoodi NN, Muncey AR, Serdiuk AA, Miller MD, Hanna MM, Laborde JM, Getting Garcia RE (2021). A Retrospective Analysis of Patients Undergoing Telemedicine Evaluation in the PreAnesthesia Testing Clinic at H. Lee Moffitt Cancer Center. Cancer Control.

[5] He Y, Aliyu A, Evans M, Luo C (2021). Health Care Cybersecurity Challenges and Solutions Under the Climate of COVID-19: Scoping Review. J Med Internet Res.

[6] Saeed H, Malik H, Bashir U, Ahmad A, Riaz S, Ilyas M, Bukhari WA, Khan MIA (2022). Blockchain technology in healthcare: A systematic review. PLoS ONE.

[7] Čapko D, Vukmirović S, Nedić N (2022) “State of the art of zero-knowledge proofs in Blockchain,” 2022 30th telecommunications forum (TELFOR), Belgrade, Serbia,1-4 10.1109/TELFOR56187.2022.9983760

[8] Bignami E, Panizzi M, Bellini V (2024). Artificial Intelligence for Personalized Perioperative Medicine. Cureus.

[9] Hayasaka T, Kawano K, Kurihara K, Suzuki H, Nakane M, Kawamae K (2021). Creation of an artificial intelligence model for intubation difficulty classification by deep learning (convolutional neural network) using face images: an observational study. J Intensive Care.

[10] Wong DT, Kamming D, Salenieks ME, Go K, Kohm C, Chung F (2004). Preadmission anesthesia consultation using telemedicine technology: a pilot study. Anesthesiology.

[11] Bhanvadia RR, Carpinito GP, Kavoussi M, Lotan Y, Margulis V, Bagrodia A, Roehrborn CG, Gahan JC, Cadeddu J, Woldu S (2022). Safety and Feasibility of Telehealth Only Preoperative Evaluation Before Minimally Invasive Robotic Urologic Surgery. J Endourol.

[12] Morau E, Chevallier T, Serrand C, Perin M, Gricourt Y, Cuvillon P (2024). Teleconsultation compared with face-to-face consultation in the context of pre-anesthesia evaluation: TELANESTH, a randomized controlled single-blind non-inferiority study. J Clin Anesth.

[13] Applegate RL, Gildea B, Patchin R, Rook JL, Wolford B, Nyirady J, Dawes TA, Faltys J, Ramsingh DS, Stier G (2013). Telemedicine pre-anesthesia evaluation: a randomized pilot trial. Telemed J E Health.

[14] Khera KD, Blessman JD, Deyo-Svendsen ME, Miller NE, Angstman KB (2022). Pre-Anesthetic Medical Evaluations: Criteria Considerations for Telemedicine Alternatives to Face to Face Visits. Health Serv Res Manag Epidemiol.

[15] Vacheron CH, Ferrier C, Morau E, Theissen A, Piriou V, Carry PY, Friggeri A (2023). Pre-anaesthesia Telephone Consultation: A Safe Alternative for Anaesthesia Assessment in Case of Repeated Low or Intermediate Risk Surgeries: A Prospective Cohort Study. Turk J Anaesthesiol Reanim.

